# Correlation between *Calpain-10* single-nucleotide polymorphisms and obstructive sleep apnea/hypopnoea syndrome with ischemic stroke in a Chinese population: A population-based study

**DOI:** 10.1097/MD.0000000000006570

**Published:** 2017-04-21

**Authors:** Wei Zhang, Zhi-Ru Zhao, Chang-Fei Dai, Rong Zhang, Jie Chen, Hui-Juan Tian, Yun-Long Wang, Ji-Hong Sun, Qiu-Fang Lian

**Affiliations:** a Department of Neurology; b Department of Cardiology, Xianyang Hospital of Yan’an University, Xianyang 712000, P.R. China.

**Keywords:** *Calpain-10*, ischemic stroke, obstructive sleep apnea-hypopnea syndrome, single-nucleotide polymorphism, SNP 19, SNP 43, SNP 44

## Abstract

**Background:**

Obstructive sleep apnea-hypopnea syndrome (OSAHS) is a common chronic disorder which is followed by various complications. *Calpain-10* belongs to a commonly expressed member of the Calpain-like cysteine protease family, which acts as risk marker for some diseases. The purpose of this study is to elucidate correlation between *Calpain-10* single-nucleotide polymorphisms (SNPs) and the incidence of OSAHS followed by ischemic stroke (IS).

**Methods:**

OSAHS patients were divided as OSAHS + IS, OSAHS, and control groups, respectively. Immunohistochemistry was performed for *Calpain-10* protein expression, polymerase chain reaction (PCR)-restriction fragment length polymorphism for detection of gene polymorphisms of SNP 43 and SNP 19, and PCR-allele specific amplification for SNP 44. Polysomnography was conducted to check the nocturnal polysomnography indicators, and also Montreal Cognitive Assessment (MoCA), Scientific Data System scores cognition and anxiety of patients, respectively. Logistic analysis was used for the risky factors for OSAHS.

**Results:**

*Calpain-10* protein expression was significantly increased in the OSAHS + IS and OSAHS groups compared with the control group. Significant differences in SNP 43 and SNP 44 genotype, and also allele frequency were observed in 3 groups, among which the OSAHS + IS group had higher SNP 43 and SNP 44 allele frequency than the control and OSAHS groups. There were differences regarding apnea-hypopnea index, minimum fingertip blood oxygen saturation (LSaO_2_ [%]), oxygen reduction index (ODI) between patients with different genotypes of SNP 43 and SNP 44 in OSAHS patients, and also GC and AT frequency in the OSAHS + IS and OSAHS groups. As compared with the OSAHS group, the MoCA scores and MoCA subitems in the OSAHS + IS group were declined, whereas the Scientific Data System scores were elevated. Additionally, GG 43 genotype, high apnea-hypopnea index, and body mass index were detected as the risk factors of OSAHS.

**Conclusion:**

These findings indicate that the *Calpain-10* SNP 43 may be related to OSAHS with IS, with SNP 43 GG genotype as a risk factor for OSAHS with IS.

## 1 Introduction

Obstructive sleep apnea-hypopnea syndrome (OSAHS) is clinically defined as a disordered breathing event that may cause a blockage or narrowing of the upper airway, which is in occurrence with symptoms such as sleepiness, and also snoring.^[1]^ OSAHS is a common chronic disorder that occurs on a similar frequency as type I diabetes and twice that of asthma. The prevalence of OSAHS in the United States is estimated up to 24% for men and 9% for women.^[2]^ The pathogenesis of OSAHS involves an interaction between ventilatory control instability and poor pharyngeal anatomy. It is also believed that OSAHS may be triggered by anatomic factors that promote pharyngeal narrowing, such as large neck circumference, cervical soft tissue, vessels, and bony structures.^[3]^ OSAHS exhibits the highest incidence rate among sleep-related diseases and is also followed by various complications among which cardiovascular diseases are the most common and severe.^[4]^ To the best of our knowledge, recent years have witnessed an intensification of the study with aims to establish the genetic contribution to the occurrence of OSAHS, and also its sequelae.^[5]^

*Calpain-10* is identified as the first type 2 diabetes mellitus gene in a genome-wide scan followed by positional cloning, and is located on chromosome 2q37, encoding *Calpain-10*, which is a commonly expressed member of the Calpain-like cysteine protease family.^[6,7]^ Functional genetic data reveals that *Calpain-10* plays an instrumental part in insulin resistance and intermediate phenotypes, including those related to adipocytes.^[8,9]^*Calpain-10* may promote the translocation of GLUT4 through reorganization of the cytoskeleton. Furthermore, the *Calpain-10* gene has been implicated in several aspects of metabolism syndrome including plasma cholesterol concentrations,^[10]^ elevated body mass index (BMI),^[11]^ hypertension,^[12]^ and hypertriglyceridemia.^[13]^ Four *Calpain-10* single-nucleotide polymorphisms (SNPs) 19 (rs3842570), 43 (rs3792267), 44 (rs2975760), and 63 (rs5030952) have been commonly investigated for their potential roles, acting as risk markers for type 2 diabetes that is a complex metabolic disorder with increased risk of cardiovascular disease.^[14]^ In accord with previous studies, this study aims to perform an association study between *Calpain-10* SNPs including SNP 43, SNP 44, and SNP 19, and also the occurrence of OSAHS and OSAHS with ischemic stroke (IS).

## 2 Materials and methods

### 2.1 Study subjects

In all, 186 OSAHS patients (OSAHS group) and 198 OSAHS patients with IS (OSAHS + IS group) were admitted into the Xianyang Hospital of Yan’an University between October 2013 and November 2015. The OSAHS was diagnosed by the American Academy of Sleep Medicine (AASM) criteria,^[15]^ and the diagnosis of OSAHS patients with IS was done according to the criteria of both OSAHS^[15]^ and IS.^[16]^ The included criteria were as follows: OSAHS patients confirmed by computed tomography (CT) and magnetic resonance imaging (MRI); OSAHS patients confirmed by polysomnogram; OSAHS patients with symptoms like sleeping in the daytime and snoring at night; and OSAHS patients with good compliance. Exclusion criteria were as follows: patients with coma, much oral secretion, and no ability to expectorate; patients with pulmonary infection; patients with mass bunamiodyl in sternum, severe aerothorax, or mediastinal emphysema; patients with acute myocardial infarction and acute left ventricular failure; patients with uncontrollable acute ear, nasitis, and nasal sinusitis infection; patients with large area of cerebral infarction and brain stem infarction; patients with glaucoma; and patients with hypertension or diabetes, or undergoing antihypertension and antidiabetic treatment. All subjects included in this study were not blood-related. Additionally, this study also recruited 240 healthy individuals as the control group, who were inquired for medical history before undergoing screening for exclusion of OSAHS using a Stardust portable sleep monitor, followed by a confirmation of normal blood pressure, heart rate, electrocardiogram, breathing state, liver and kidney function, and hemogram. The clinical trial regime was approved by the Ethics Committee of Xianyang Hospital of Yan’an University and strictly complied with the Ethical Guidelines for Biomedical Research on Human Subjects in the Declaration of Helsinki. All subjects or their legal representatives signed informed contents and well understood the trail contents, processes, and possible side-effects.

### 2.2 Information collection and index detection

Baseline data of all subjects were collected, including sex, age, BMI, diabetes history, hypertension, and smoking. Each fasting subject underwent the extraction of peripheral venous blood (10 mL) in the morning, and 5 mL of the extracted blood was put into heparinized tubes. After 20 minutes of centrifugation (3000 revolutions/min), the blood sample was packed in Eppendorf tubes. Enzyme method was used to detect triacylglycerol (TG), high-density lipoprotein cholesterol (HDL-c) and low-density lipoprotein cholesterol (LDL-c) by Hitachi 7600 automatic biochemical analyzer (Hitachi High-Technologies Corporation, Tokyo, Japan). The remaining 5 mL of peripheral venous blood was put in ethylene diamine tetra acetic acid (EDTA) tubes and reserved at −80°C in a refrigerator for genomic DNA extraction and further experiments.

### 2.3 Enzyme-linked immunosorbent assay

Enzyme-linked immunosorbent assay (ELISA) was conducted for the Calpain-10 protein expression in the peripheral blood both in the case and control groups. The enzyme-labeled plate coated by antibodies were taken out, and in its wells added with standard samples and samples to be tested of various concentrations, respectively, which were then incubated at 37°C for 2 hours. After the solution elimination, 0.1 mL of biotinylated antibodies were added in each well at 37°C for 30 minutes, followed by phosphate buffer saline (PBS) washing for 5 times. Then 0.1 mL of tetramethylbenzidine (TMB) for each well was used for developing at 37°C for 20 minutes and TMB stopping solution in the same dosage for stopping reaction. The optical density (OD) in the enzyme-labeled plate was detected at the wavelength of 450 nm. The ELISA kit was purchased from the Shanghai Ximei Biotechnology Co. Ltd (ELX800, Shanghai, China).

### 2.4 Monitor index of sleep apnea

All subjects were prohibited against alcohol, coffee, tranquilizer, and hypnotic within 24 hours before monitoring, and then they continuously received sleep apnea monitoring with no less than 7 hours by a polysomnography (PSG; Somno-star; Sensormedics, Anheim, CA) at night in a sleep laboratory. Monitor index of sleep apnea included: apnea-hypopnea index (AHI), figure arterial O_2_ saturation (SaO_2_) <90%, oxygen reduction index (ODI), lowest SaO_2_ (LSaO_2_%), mean SaO_2_ (MSaO_2_%), the proportion (%) of stage I sleep and stage II sleep (S1 + S2 sleep) time in the total sleep time, the proportion (%) of stage III sleep and stage IV sleep (S3 + S4 sleep) time in the total sleep time, and the proportion (%) of rapid eye movement (REM) sleep time in the total sleep time.

### 2.5 Polymerase chain reaction-restriction fragment length polymorphism and PCR allele-specific amplification (ASA)

A total of 2 mL of EDTA anticoagulant, genomic DNA from peripheral blood according to the conventional phenol/chloroform/isopropanol method^[17]^ were extracted using EPPendorf UV spectrophotometry to measure DNA concentration and normalize it to 50 ng/mL. The 3 SNP primers of *Calpain-10* gene were synthesized by Shanghai Boya biological Co. Ltd (Shanghai, China) as shown in Table 1. The PCR reaction system^[14]^ included 2.5 μL of 10× PCR buffer solution, 11.3 μL of ddH_2_O, 2 μL of dNTP, 0.2 μL (5 U/μL) of Taq polymerase, 2 μL of template DNA, 1 μL of the upstream and downstream primers, and 0.75 μL of R2 and 0.25 μL of L primer in the SNP44 locus R1 primer. The PCR conditions are as follows: predenaturation at 95°C for 5 minutes, 30 cycles of denaturation at 95°C for 30 seconds, annealing at 55°C for 30 seconds, and amplification at 72°C for 30 seconds, and final extension at 72°C for 10 minutes. The products of SNP 43 were identified by 2% gel electrophoresis after digested by NsiI incision enzyme in water bath at 37°C for 16 hours. However, the products of SNP 19 and SNP 44 are directly identified by 2% gel electrophoresis.

**Table 1:**
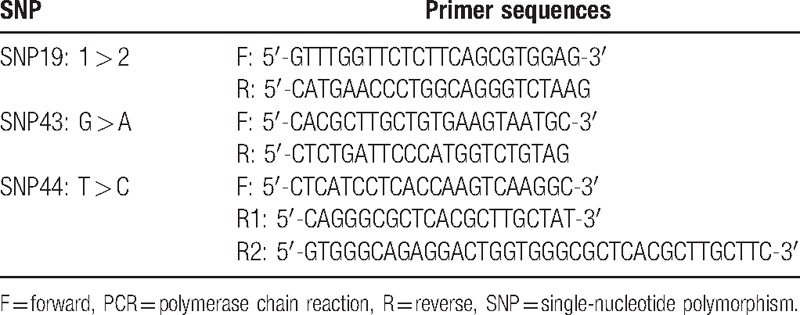
PCR-amplified primer of *Calpain-10* gene SNP 19, SNP 43, and SNP 44.

### 2.6 Restriction enzyme digestion and electrophoresis

**Figure 1:**
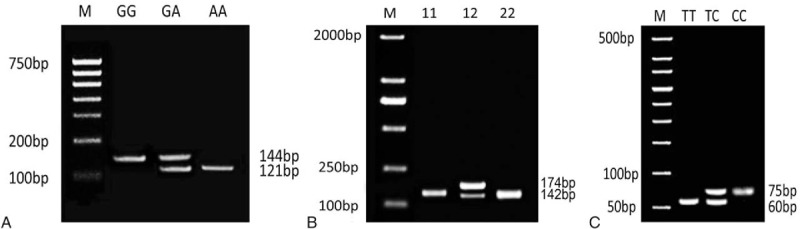
Electrophoresis of *Calpain-10* gene SNP 43, SNP 19, and SNP 44. A, SNP 43; B, SNP 19; C, SNP 19. SNP = single-nucleotide polymorphism.

The products of PCR SNP43 were identified by NsiI enzyme. After restriction enzyme treatment, 3 kinds of genotypes were produced: wild-type GG for 144 bp, heterozygous mutation type for 144, 121, and 23 bp, and pure mutant type AA for 121 and 23 bp. SNP19 was the insertion deletion polymorphism, and its PCR products after electrophoresis were directly divided into 3 types: wild-type 11 for 142 bp, heterozygous mutant 12 for 174 and 142 bp, and pure mutant type 22 for 174 bp. PCR SNP44 products was directly assigned into 3 kinds of genotypes after electrophoresis: wild-type TT for 60 bp, heterozygous mutant TC for 60 and 75 bp, and pure mutant CC for 75 bp (as shown in Fig. 1).

### 2.7 Assessment of cognition and anxiety

All patients were assessed using the Montreal cognitive function rating scale (MoCA)^[18]^ in the same order. The total scores of MoCA ≥26 represented normal cognitive function. Depression Scale Self-rating (Scientific Data System [SDS])^[19]^ was used to evaluate the anxiety of the study objects, with SDS <50 meaning no anxiety, 50 to 59 meaning mild anxiety, 60 to 69 meaning severe anxiety, and over 70 meaning severe anxiety.

### 2.8 Statistical analysis

SPSS 21 statistical software (SPSS Inc., Chicago, IL) was used to statistically analyze the data. The measurement data were expressed by mean ± standard deviation (SD), and before data analysis, normality and homogeneity of variance tests were conducted. If the distribution and variance were normally presented, the *t* test was used in the 2 groups for comparison and 1-way analysis of variance (ANOVA) for single-factor analysis of variance for groups, and tested by SNK-q test. If not, the nonparametric Kruskal-Wallis h rank-sum test was conducted, and Mann-Whitney *U* test was conducted for group comparisons. The count data were expressed by constituent ratio or rate using chi-square test. Relative risk of genotype was expressed by odds ratio (OR) and 95% confidence interval (CI). Hardy-Weinberg equilibrium method was used to detect group representation of samples. SHEsis software was utilized for haploid analysis and logistic regression method for the risk factors for OSAHS with IS, with *P* < 0.05 meaning statistically difference.

## 3 Results

### 3.1 Clinical data of subjects among the OSAHS, OSAHS + IS, and control groups

As shown in Table 2, there were statistical differences in AHI, BMI among the OSAHS, OSAHS + IS, and control groups (all *P* < 0.05), whereas no significant difference of age, sex, smoking history, history of diabetes, history of hypertension, and 3 acyl glycerin (TG), HDL-c, LDL-c content ratio was found among the OSAHS, OSAHS + IS, and control groups (all *P* > 0.05).

**Table 2:**
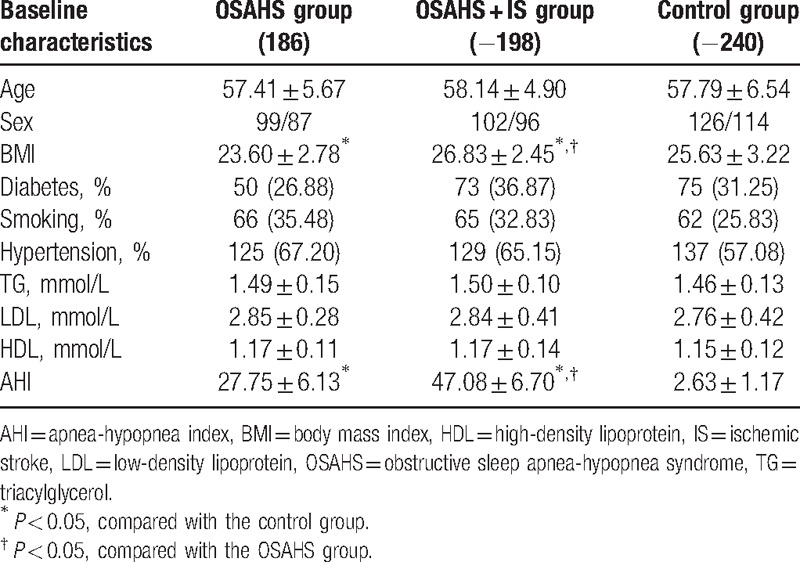
Comparisons of baseline characteristics of subjects among the OSAHS group, OSAHS + IS group, and control group.

### 3.2 The Calpain-10 protein expression in serum among 3 groups

**Figure 2:**
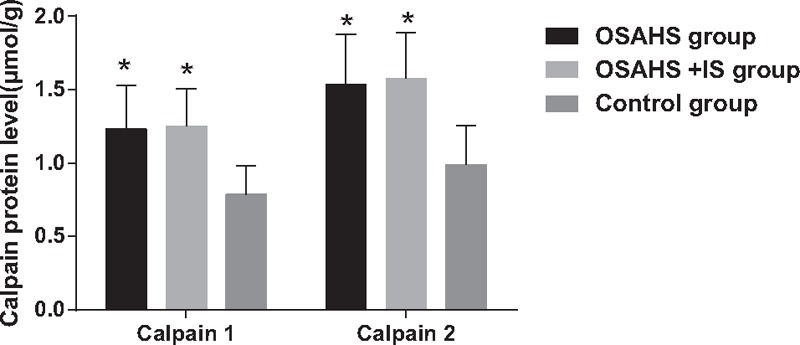
Calpain-10 protein expressions in serum among 3 groups. IS = ischemic stroke, OSAHS = obstructive sleep apnea-hypopnea syndrome. (∗) *P* < 0.05, compared with the control group.

The Calpain-10 protein expression in serum among 3 groups was detected by ELISA, as shown in Fig. 2. Compared with the control group, the Calpain-10 protein expression was increased significantly in the OSAHS and OSAHS + IS groups (*P* < 0.05).

### 3.3 Genotype and allele frequency distribution of *Calpain-10* SNPs among the OSAHS, OSAHS + IS, and control groups

The difference between the actual frequency and the theoretical frequency of SNPs *Calpain-10* genotypes was assessed by the goodness-of-fit chi-square test. This study included a total of 208 subjects. After inspection, the results showed that there were no significant differences between observed value and estimated value of the genotype distribution frequency of Calpain-10 SNP 43, SNP 44, and SNP 19 (*P* > 0.05), which indicated that the samples were from a large group with randomly assigned equilibrium (as shown in Table 3).

**Table 3:**
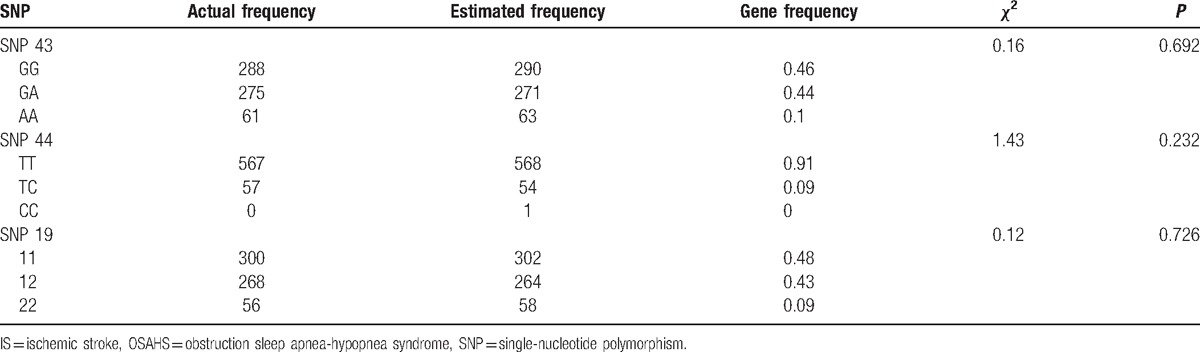
The results of Hardy-Weinberg equilibrium analysis for *Calpain-10* gene SNP 19, SNP 43, and SNP 44.

There were significant differences of SNP 43 and SNP 44 genotype and allele frequency between the OSAHS + IS and OSAHS groups, and between the OSAHS + IS and control groups (*P* < 0.05), whereas no significant difference was shown between the control and OSAHS groups (*P* > 0.05). SNP 43 G and SNP 44 C allele frequencies in OSAHS + IS group were significantly higher than those in the control and OSAHS groups (all *P* < 0.05). However, there was no remarkable difference regarding SNP 19 genotype and allele frequency among the OSAHS, OSAHS + IS, and control groups (*P* > 0.05) (as shown Table 4).

**Table 4:**
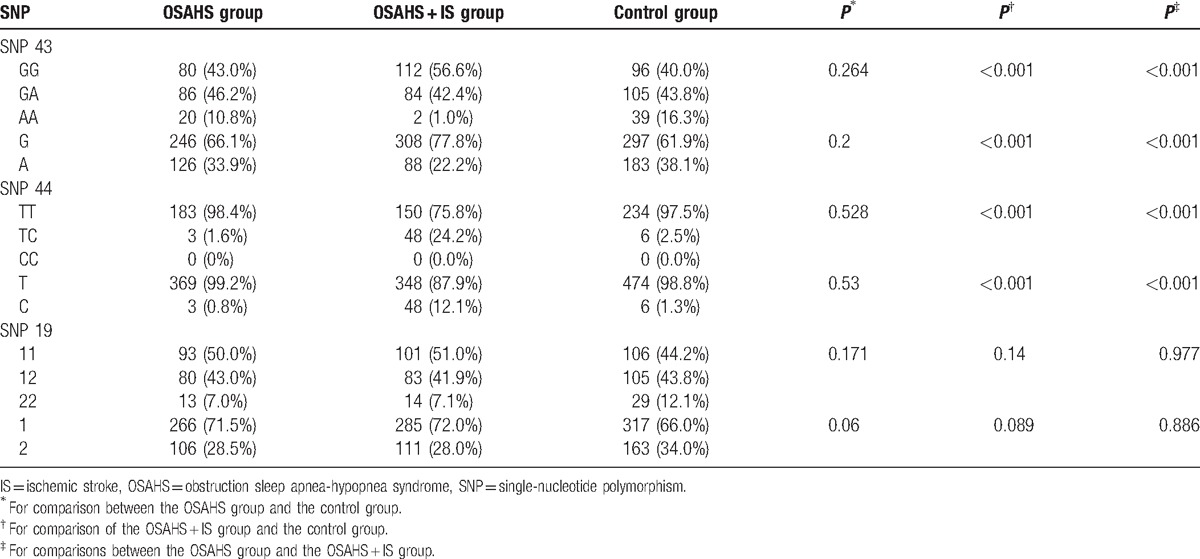
Comparisons of genotype and allele frequency of *Calpain-10* gene SNP 44, SNP 19, and SNP 43 among the OSAHS, OSAHS + IS, and control groups.

### 3.4 Comparisons on sleep respiratory monitoring indexes of OSAHS patients with different genotypes of *Calpain-10* SNPs

Sleep respiratory monitoring indexes of patients with different OSAHS genotypes detected by the PSG showed that there were significant differences concerning AHI, LSaO_2_, and ODI (all *P* < 0.05), but no differences in MSaO_2_, S1 + S2 (%), S3 + S4 (%), and REM (%) among patients with different SNP 43 and SNP 44 genotypes (all *P* > 0.05). The differences of sleep respiratory monitoring indexes were not significant (*P* > 0.05) among the patients with various SNP 19 genotypes (as shown in Table 5).

**Table 5:**
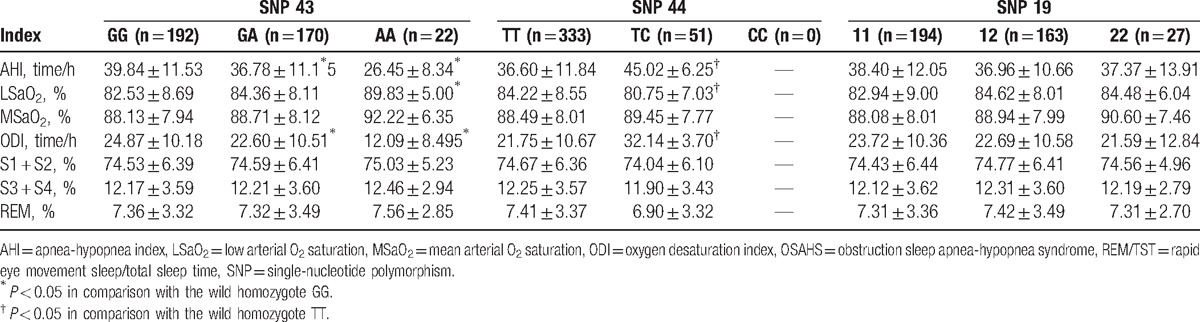
Comparisons on sleep respiratory monitoring indexes of OSAHS patients with different genotypes of *Calpain*-10 SNPs.

### 3.5 Haplotype analysis of SNP 43 and SNP 44 in *Calpain-10* gene

The SHEsis software was used in the linkage disequilibrium and haplotype analysis of *Calpain-10* SNP 43 and SNP 44 in the OSAHS and OSAHS + IS groups. During haplotype analysis, haplotypes whose frequencies were no more than 3% were deleted, and the results showed that the differences of GC and AT haplotype between the OSAHS and OSAHS + IS groups were statistically significant (*χ*^**2**^ = 39.609, *P* *<* 0.001; *χ*^**2**^ = 12.949, *P* *<* 0.001) (as shown in Table 6).

**Table 6:**
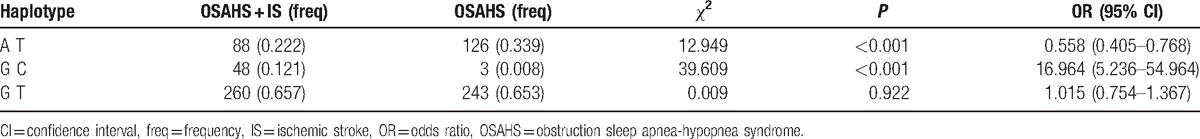
Haplotype analysis of *Calpain-10* gene SNP 44, SNP 19, and SNP 43 among the OSAHS group and OSAHS + IS groups.

### 3.6 Comparisons of cognition and anxiety between the OSAHS and OSAHS + IS groups

Patients underwent SDS and MoCA scale evaluation after admission to the hospital. Compared with the OSAHS group, the MoCA scale scores were significantly lower, whereas the SDS scores were significantly higher in the OSAHS + IS group (both *P* < 0.05). The MoCA sub items in OSAHS + IS group such as attention and calculation, abstract, visual space, and executive function and delayed recall significantly decreased (*P* < 0.05). No significant difference was found in other subitems (*P* > 0.05), as shown in Table 7.

**Table 7:**
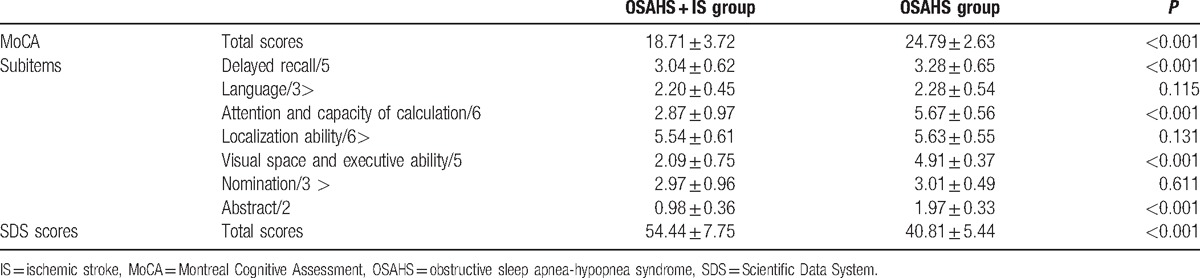
Comparisons of cognition and anxiety between the OSAHS and OSAHS + IS groups (mean ± standard deviation).

### 3.7 Logistic regression analysis for risk factors of OSAHS with IS

Using *Calpain-10* SNP 43 and SNP 44, BMI, AHI in the OSAHS and OSAHS + IS groups as independent variables, OSAHS + IS as dependent variable, unconditioned logistic regression analysis was performed to estimate regression coefficient and calculate OR with 95% CI for risk factor of OSAHS with IS (OR > 1). As shown in Table 8, SNP 43 GG genotypes, high AHI, and high BMI were the risk factors of OSAHS with IS (OR > 1, *P* < 0.05).

**Table 8:**
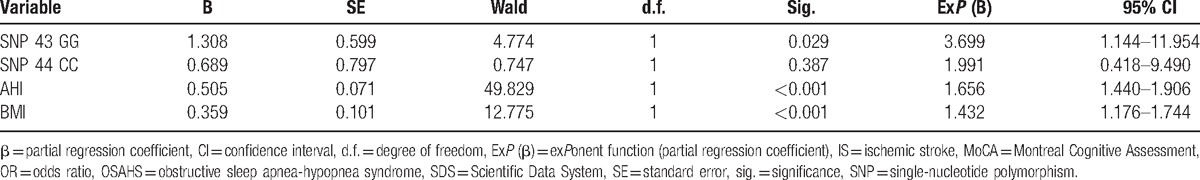
Unconditioned logistic regression analysis for risk factors of OSAHS with IS.

## 4 Discussion

The study was designed to elucidate association between *Calpain-10* gene polymorphisms and the occurrence of OSAHS and OSAHS with IS. SNP 63 was identified as minor allele with the lowest allele frequency, always selected for some rare polymorphism studies; thus it was excluded in this study.^[20,21]^ Therefore, we demonstrated the positive association between *Calpain-10* SNP 43 and SNP 44 polymorphisms, and the risk of OSAHS and OSAHS with IS.

The key findings from this study indicated that the frequency of SNP 43 GG genotype and SNP 44 TC genotype in the OSAHS and OSAHS + IS groups were remarkably higher than that in the case group. Calpains are a very common family of the calcium-dependent cysteine proteases which are always involved in a large range of differentiation processes and cell regulatory.^[22]^ It was reported that Calpains played a pivotal role in neurodegeneration, cell motility, and synaptic plasticity, among which mu-calpain (calpain-1) and m-calpain (calpain-2) were 2 major calpain isoforms shown in brain.^[23]^ Calpain-10 protein, as intracellular Ca (2+)-dependent cysteine protease, exerts effects on regulation of thermogenesis, pancreatic β-cell function, and glucose metabolism, and several sites of Calpain-10 polymorphism are researched for their potential markers for the metabolic syndrome and type 2 diabetes.^[6]^ It is sited in mitochondria, which is of importance in mitochondrial homeostasis, implicated in diabetes-induced renal dysfunction, indicating that loss of Calpain-10 induced by glucose in vivo leads to organ failure and renal cell apoptosis via collections of mitochondrial dysfunction and mitochondrial calpain-10 substrates.^[24]^ To our knowledge, there is a connection between the severity of OSAHS and both damaged glucose tolerance, and also insulin resistance, suggesting that obstructive sleep apnea-related diseases are risk factors for the occurrence of cardiovascular disease, such as type 2 diabetes.^[25]^ Also, the occurrence rate of obstructive sleep apnea-related diseases was found significantly higher in the patients with type 2 diabetes.^[26]^ These data have proposed the presumption of type 2 diabetes as a risk factor for OSAHS, arising with a bidirectional fashion, and *Calpain-10* was defined as the first gene influencing the risk of type 2 diabetes by positioning cloning and the possibility of association between SNP 43 and SNP 44 polymorphisms, and transcriptional regulation *Calpain-10* expression has been raised.^[14]^ A study performed by Ling et al^[27]^ has reported that the *Calpain-10* mRNA level was increased by 64% in pancreatic islets from patients with type 2 diabetes in comparison with nondiabetic individuals. Horikawa et al^[28]^ also discovered that mutation in *Calpain-10* gene has been linked to a 3-fold increased risk of type 2 diabetes in Mexican-Americans, and also in Northern European populations. It was also believed that variation of the *Calpain-10* gene (SNP 43 GG and SNP 44 TT) had connections with elevated levels of total cholesterol.^[13,29]^ Taken together, our study assumed that SNP 43 and SNP 44 polymorphisms of *Calpain-10* gene might affect the variance in *Calpain-10* mRNA level so as to increase the risk of cardiovascular diseases, ultimately contributing to the development of OSAHS. In our study, however, there was no significant difference in SNP 19 genotype and allele frequency between the OSAHS, OSAHS + IS, and control groups. In the study by Zaharna et al,^[30]^ they uncovered that, in the donors with type 2 diabetes, the total cholesterol levels were higher in the SNP 19 22 genotype than those in 12 or 11 genotype, which was significantly different from our results. Nevertheless, the above positive data were also changed by the studies conducted by Carlsson et al^[13]^ and Daimon et al.^[29]^ This difference in results could be due to the limited studies that investigated total cholesterol, and also triglyceride levels and their associations with *Calpain-10,* which needs further investigation.

Additionally, there were significant differences in these monitor indexes of sleep apnea between the OSAHS patients and OSAHS patients with IS. The severity of OSAHS is commonly measured by the AHI that reflects the number of AHI per hour of sleep, with 5/h regarded as normal, AHI 5 to 15/h as mild, AHI 15 to 29/h as moderate, and AHI 30/h as severe.^[26]^ In our study, we found that the OSAHS patients with IS show increased AHI compared with the OSAHS patients, possibly suggesting that OSAHS with IS would deteriorate OSAHS. The ODI refers to the number of events per hour in which oxygen saturation declines by 4% or more. Interestingly, hypopnea implicates a reduction of airflow by 50% to 80% for at least 10 seconds related to either oxygen desaturation of at least 4% or arousals. Our results also demonstrated that the OSAHS patients with IS had increased ODI compared with the OSAHS patients. Consistent with our study, Park et al^[31]^ supported the possibility of the severe OSAHS patients with greater oxygen desaturation. Also, our study revealed that the OSAHS patients with IS exhibit decreased LSaO_2_ and MSaO_2_. As reported in the study performed by Ursavas et al,^[32]^ the patients with OSAHS had remarkable lower MSaO_2_, but longer time of SaO_2_ <90% than the healthy controls. Also, there were significant differences in AHI, LSaO_2_, MSaO_2_, ODI, time of SaO_2_ <90%, and REM/TST among the SNP 43 GG, GA, and AA, and between the SNP 44 TT and TC, suggesting that SNP 43 GG and SNP 44 TG is linked to OSAHS.

In conclusion, the logistic regression analysis indicated that SNP 43 AA, SNP 44 TC, and larger neck circumference were the risk factors of OSAHS and OSAHS with IS. Collectively, *Calpain-10* SNP 43 and SNP 44 polymorphisms were correlated with the risk of OSAHS and OSAHS with IS. Disease association with certain variants of *Calpain-10* found in certain populations may not be applicable for other populations due to detecting the expected variants in only part of all populations. Increased or decreased frequency of particular genotypes depends on genetic and environmental factors, such as linkage disequilibrium, founder effect together with selection. Apart from this, our study consisted of a small sample size, since it is better to recruit a large sample size in genetic association studies. Thus, further in-depth researches will be performed to explore the mechanism of *Calpain-10* SNPs on the influencing factors, such as total cholesterol and triglycerides in cardiovascular diseases and metabolism syndromes.

## Acknowledgment

We thank the reviewers for critical comments.

## References

[1] Kielb SA Ancoli-Israel S Rebok GW Cognition in obstructive sleep apnea-hypopnea syndrome (OSAS): current clinical knowledge and the impact of treatment. Neuromolec Med 2012;14:180–93. 10.1007/s12017-012-8182-1 PMC3823054 22569877

[2] Sankri-Tarbichi AG Obstructive sleep apnea-hypopnea syndrome: etiology and diagnosis. Avicenna J Med 2012;2:3–8. 23210013 10.4103/2231-0770.94803 PMC3507069

[3] De Backer W Obstructive sleep apnea/hypopnea syndrome. Panminerva Med 2013;55:191–5. 23676959

[4] Pack AI Gislason T Obstructive sleep apnea and cardiovascular disease: a perspective and future directions. Prog Cardiovasc Dis 2009;51:434–51. 19249449 10.1016/j.pcad.2009.01.002

[5] Kent BD Ryan S McNicholas WT The genetics of obstructive sleep apnoea. Curr Opin Pulm Med 2010;16:536–42. 20814305 10.1097/MCP.0b013e32833ef7fe

[6] Perez-Martinez P Delgado-Lista J Garcia-Rios A Calpain-10 interacts with plasma saturated fatty acid concentrations to influence insulin resistance in individuals with the metabolic syndrome. Am J Clin Nutr 2011;93:1136–41. 21389182 10.3945/ajcn.110.010512

[7] Hanis CL Boerwinkle E Chakraborty R A genome-wide search for human non-insulin-dependent (type 2) diabetes genes reveals a major susceptibility locus on chromosome 2. Nat Genet 1996;13:161–6. 8640221 10.1038/ng0696-161

[8] Saez ME Gonzalez-Sanchez JL Ramirez-Lorca R The CAPN10 gene is associated with insulin resistance phenotypes in the Spanish population. PLoS One 2008;3:e2953. 18698425 10.1371/journal.pone.0002953 PMC2495037

[9] Turner MD Cassell PG Hitman GA Calpain-10: from genome search to function. Diabetes Metab Res Rev 2005;21:505–14. 16028216 10.1002/dmrr.578

[10] Wu B Takahashi J Fu M Variants of calpain-10 gene and its association with type 2 diabetes mellitus in a Chinese population. Diabetes Res Clin Pract 2005;68:155–61. 15860244 10.1016/j.diabres.2004.09.015

[11] Shima Y Nakanishi K Odawara M Association of the SNP-19 genotype 22 in the calpain-10 gene with elevated body mass index and hemoglobin A1c levels in Japanese. Clin Chim Acta 2003;336:89–96. 14500039 10.1016/s0009-8981(03)00320-6

[12] Chen SF Lu XF Yan WL Variations in the calpain-10 gene are associated with the risk of type 2 diabetes and hypertension in northern Han Chinese population. Chin Med J (Engl) 2007;120:2218–23. 18167206

[13] Carlsson E Fredriksson J Groop L Variation in the calpain-10 gene is associated with elevated triglyceride levels and reduced adipose tissue messenger ribonucleic acid expression in obese Swedish subjects. J Clin Endocrinol Metab 2004;89:3601–5. 15240652 10.1210/jc.2003-032105

[14] Bodhini D Radha V Ghosh S Association of calpain 10 gene polymorphisms with type 2 diabetes mellitus in Southern Indians. Metabolism 2011;60:681–8. 20667559 10.1016/j.metabol.2010.07.001

[15] Robinson SE Martin RM Davis TR The effect of acetylcholine depletion on behavior following traumatic brain injury. Brain Res 1990;509:41–6. 2306637 10.1016/0006-8993(90)90306-v

[16] Xie RM Chen HX Xie YM Effects of integrative medicine protocols on the improvement of neural function deficit and disability outcomes in patients with acute ischemic cerebral stroke. Zhongguo Zhong Xi Yi Jie He Za Zhi 2011;31:1175–80. 22013790

[17] Schutz J Backmann L Terwort H Carotid angiography in the diagnosis of skull-brain injuries. Radiologe 1972;12:294–7. 4642065

[18] Nasreddine ZS Phillips NA Bedirian V The Montreal Cognitive Assessment, MoCA: a brief screening tool for mild cognitive impairment. J Am Geriatr Soc 2005;53:695–9. 15817019 10.1111/j.1532-5415.2005.53221.x

[19] Li Y Zeng Y Zhu W Path model of antenatal stress and depressive symptoms among Chinese primipara in late pregnancy. BMC Pregnancy Childbirth 2016;16:180. 27439302 10.1186/s12884-016-0972-2 PMC4955111

[20] Polychronakos C Common and rare alleles as causes of complex phenotypes. Curr Atheroscler Rep 2008;10:194–200. 18489846 10.1007/s11883-008-0031-1

[21] Kurzawski M Dziewanowski K Kedzierska K Association of calpain-10 gene polymorphism and posttransplant diabetes mellitus in kidney transplant patients medicated with tacrolimus. Pharmacogenom J 2010;10:120–5. 10.1038/tpj.2009.44 19752882

[22] Liu W Apagyi K McLeavy L Expression and cellular localisation of calpain-like proteins in *Trypanosoma brucei*. Mol Biochem Parasitol 2010;169:20–6. 19766148 10.1016/j.molbiopara.2009.09.004

[23] Zadran S Jourdi H Rostamiani K Brain-derived neurotrophic factor and epidermal growth factor activate neuronal m-calpain via mitogen-activated protein kinase-dependent phosphorylation. J Neurosci 2010;30:1086–95. 20089917 10.1523/JNEUROSCI.5120-09.2010 PMC2820881

[24] Covington MD Schnellmann RG Chronic high glucose downregulates mitochondrial calpain 10 and contributes to renal cell death and diabetes-induced renal injury. Kidney Int 2012;81:391–400. 22012129 10.1038/ki.2011.356

[25] Botros N Concato J Mohsenin V Obstructive sleep apnea as a risk factor for type 2 diabetes. Am J Med 2009;122:1122–7. 19958890 10.1016/j.amjmed.2009.04.026 PMC2799991

[26] Rajan P Greenberg H Obstructive sleep apnea as a risk factor for type 2 diabetes mellitus. Nat Sci Sleep 2015;7:113–25. 26491377 10.2147/NSS.S90835 PMC4599645

[27] Ling C Groop L Guerra SD Calpain-10 expression is elevated in pancreatic islets from patients with type 2 diabetes. PLoS One 2009;4:e6558. 19688040 10.1371/journal.pone.0006558 PMC2719809

[28] Horikawa Y Oda N Cox NJ Genetic variation in the gene encoding calpain-10 is associated with type 2 diabetes mellitus. Nat Genet 2000;26:163–75. 11017071 10.1038/79876

[29] Daimon M Oizumi T Saitoh T Calpain 10 gene polymorphisms are related, not to type 2 diabetes, but to increased serum cholesterol in Japanese. Diabetes Res Clin Pract 2002;56:147–52. 11891023 10.1016/s0168-8227(01)00372-2

[30] Zaharna MM Abed AA Sharif FA Calpain-10 gene polymorphism in type 2 diabetes mellitus patients in the Gaza Strip. Med Princ Pract 2010;19:457–62. 20881413 10.1159/000320304

[31] Park DH Shin CJ Hong SC Correlation between the severity of obstructive sleep apnea and heart rate variability indices. J Korean Med Sci 2008;23:226–31. 18437004 10.3346/jkms.2008.23.2.226 PMC2526439

[32] Ursavas A Ilcol YO Nalci N Ghrelin, leptin, adiponectin, and resistin levels in sleep apnea syndrome: role of obesity. Ann Thorac Med 2010;5:161–5. 20835311 10.4103/1817-1737.65050 PMC2930655

